# Analysis of mortality by cardiovascular disease subgroups in Brazil before and during the COVID-19 pandemic (2000–2022) by sex and age group

**DOI:** 10.1590/1980-549720250033

**Published:** 2025-06-27

**Authors:** Cicero Emanoel Alves Leite, Raphael Mendonça Guimarães, Andréa Sobral

**Affiliations:** I Fundação Oswaldo Cruz, Sérgio Arouca National School of Public Health, Postgraduate Program in Public Health and Environment – Rio de Janeiro (RJ), Brazil.; II Fundação Oswaldo Cruz, Sérgio Arouca National School of Public Health, Department of Social Sciences – Rio de Janeiro (RJ), Brazil.; III Fundação Oswaldo Cruz, Sérgio Arouca National School of Public Health, Department of Endemic Diseases Samuel Pessoa – Rio de Janeiro (RJ), Brazil.

**Keywords:** Time series studies, Comorbidity, Non-communicable diseases, COVID-19, Mortality

## Abstract

**Objective:**

To analyze the effect of the COVID-19 pandemic on mortality by cardiovascular disease (CVD) subgroups in Brazil, considering sex and age group.

**Methods:**

Ecological time-series study with secondary data from the Mortality Information System for the period 2000-2022. Age-adjusted mortality rates per 100,000 inhabitants were calculated and joinpoint regression models were used to estimate rate trends.

**Results:**

Mortality from CVD in Brazil between 2000 and 2022 showed a decrease in ischemic heart disease (IHD) and cerebrovascular disease (CBVD), while other CVD showed an unstable behavior, increasing after 2020. The reduction in IHD was more pronounced until 2020, decelerating thereafter. CBVD continued to decline but at a slower pace. Other CVD, after a downward trend, showed a significant increase (+3.8% per year), possibly because of the pandemic. Women had a greater reduction in mortality, especially due to IHD. Intermediate age groups (40-59 years) showed a decrease until 2020 but recorded increases after this period, reinforcing the impact of the pandemic. In 2021, all subgroups showed excess mortality: 8% for CBVD and IHD, and 15% for other CVD. In 2022, this pattern intensified, with values of 16% (CBVD), 18% (IHD) and 23% (other CVD).

**Conclusion:**

The COVID-19 pandemic significantly impacted CVD mortality in Brazil, changing trends according to age group, sex and disease subgroup. The study points to an inflection in mortality rates during the pandemic, highlighting the need for further investigations to understand the mechanisms involved.

## INTRODUCTION

Cardiovascular diseases (CVD) are responsible for approximately 17.9 million deaths per year, the leading cause of mortality worldwide^1^ and are associated with sociodemographic, health and lifestyle factors^2^. In Brazil in 2019, ischemic heart disease (IHD) and cerebrovascular diseases (CBVD) were responsible for 21.4% of all deaths overall^3^. In 2021, the prevalence of CVD was 6.9% in the Brazilian population, being higher among men (7.6%) than women (6.3%)^4^. In addition to the effect on mortality, this group of diseases reduces life expectancy and causes disabilities^5^.

However, events with a major impact on public health, such as the COVID-19 pandemic, can change these trends, either through direct effects of the SARS-CoV-2 infection or through indirect impacts, such as restrictions on access to health services and changes in population behavior.

Infectious and parasitic diseases became the leading cause of death due to the high burden of COVID-19 in 2021^5^. People with comorbidities, including CVD, put a strong strain on the health system because they are at increased risk of death from COVID-19^6^. In Brazil, health services were reorganized during the pandemic to mitigate the risk of contagion and ensure care for patients with COVID-19, which affected the care of people with comorbidities^7^.

Accordingly, understanding how the pandemic influenced mortality from different CVD subgroups, stratified by sex and age group, is essential to assess possible setbacks in the advances made in recent decades and to guide public mitigation policies. Thus, this study investigated the effect of the COVID-19 pandemic on mortality from CVD subgroups in Brazil, considering sex and age group between 2000 and 2022.

## METHODS

This study is a time series analysis, addressing mortality rates in CVD subgroups over the period from 2000 to 2022. For this analysis, the following CVD subgroups were defined based on the 10th edition of the International Classification of Diseases and Related Health Problems (ICD-10): IHD, CBVD and other CVD (Chart 1).

**Chart 1:** Category of the 10th edition of the International Classification of Diseases and Related Health Problems (ICD-10) according to the subgroup of cardiovascular diseases (CVD).

CVD subgroup	Category ICD-10
Ischemic heart diseases (IHD)	I20 Angina pectoris
	I21 Acute myocardial infarction
	I22 Recurrent myocardial infarction
	I23 Some current complications following acute myocardial infarction
	I24 Other acute ischemic heart diseases
	I25 Chronic ischemic heart disease
Cerebrovascular diseases (CBVD)	I60 Subarachnoid hemorrhage
	I61 Intracerebral hemorrhage
	I62 Other non-traumatic intracranial hemorrhages
	I63 Cerebral infarction
	I64 Stroke, not specified as ischemic hemorrhage
	I65 Occlusion and stenosis of precerebral arteries not resulting in cerebral infarction
	I66 Occlusion and stenosis of cerebral arteries not resulting in cerebral infarction
	I67 Other cerebrovascular diseases
	I68 Cerebrovascular disorders in diseases classified elsewhere
	I69 Sequelae of cerebrovascular diseases
Other CVD	I00 Rheumatic fever without mention of cardiac involvement
	I01 Rheumatic fever with cardiac involvement
	I02 Rheumatic chorea
	I05 Rheumatic diseases of mitral valve
	I06 Rheumatic diseases of aortic valve
	I07 Rheumatic diseases of tricuspid valve
	I08 Multiple valve diseases
	I09 Other rheumatic diseases of heart
	I10 Essential hypertension
	I11 Hypertensive heart disease
	I12 Hypertensive kidney disease
Other CVD	I13 Hypertensive heart and kidney disease
	I15 Secondary hypertension
	I26 Pulmonary embolism
	I27 Other form of pulmonary heart disease
	I28 Other disease of pulmonary vessels
	I30 Acute pericarditis
	I31 Other disease of pericardium
	I32 Pericarditis in diseases classified elsewhere
	I33 Acute and subacute endocarditis
	I34 Nonrheumatic disorders of mitral valve
	I35 Non-rheumatic aortic valve disorders
	I36 Non-rheumatic tricuspid valve disorders
	I37 Pulmonary valve disorders
	I38 Endocarditis of unspecified valve
	I39 Endocarditis and cardiac valvular disorders in diseases classified elsewhere
	I40 Acute myocarditis
	I41 Myocarditis in diseases classified elsewhere
	I42 Cardiomyopathies
	I43 Cardiomyopathy in diseases classified elsewhere
	I44 Atrioventricular and left bundle branch block
	I45 Other conduction disorders
	I46 Cardiac arrest
	I47 Paroxysmal tachycardia
	I48 Atrial flutter and fibrillation
	I49 Other cardiac arrhythmias
	I50 Heart failure
	I51 Complications of heart disease and ill-defined cardiac diseases
	I52 Other cardiac conditions in diseases classified elsewhere
	I70 Atherosclerosis
	I71 Aneurysm and dissection of aorta
	I72 Other aneurysms
	I73 Other peripheral vascular diseases
	I74 Arterial embolism and thrombosis
	I77 Other disorders of arteries and arterioles
	I78 Disease of capillaries
	I79 Disorders of arteries, arterioles and capillaries in diseases classified elsewhere
	I80 Phlebitis and thrombophlebitis
	I81 Portal vein thrombosis
	I82 Other venous embolism and thrombosis
	I83 Varicose veins of lower limbs
	I84 Hemorrhoids
	I85 Esophageal varices
	I86 Varicose veins of other sites
	I87 Other disorders of veins
	I88 Nonspecific lymphadenitis
	I89 Other non-infectious disorders of lymphatic vessels and lymph nodes
	I95 Hypotension
	I97 Disorders of the circulatory system subsequent to procedures not classified elsewhere
	I98 Other disorders of circulatory system in diseases classified elsewhere
	I99 Other and unspecified disorders of circulatory system

The data were obtained from the Mortality Information System, made publicly available by the Department of Information Technology of the Unified Health System, and categorized by CVD subgroups, differentiating the variables sex, year of death and age categorized by age group. The population base used was that of projections and back projections carried out by the Brazilian Institute of Geography and Statistics for each location and year.

To analyze the trends, we performed segmented regression using the Joinpoint Regression 5.0 program, which identifies inflections in the temporal trend over time. Annual rates adjusted for age were determined, assuming constant variance (homoscedasticity). The model was selected using the weighted Bayesian information criterion. In addition, confidence intervals (CI) were used to estimate the annual percent change (APC) and the average annual percent change (AAPC) and to determine change points using the empirical quantile method. The trends (APC and AAPC) were classified as stable (p³0.05 and positive or negative regression coefficient), increasing (p<0.05 and positive coefficient) and decreasing (p<0.05 and negative coefficient).

To analyze excess mortality in each population group, the number of deaths expected for the years of the COVID-19 pandemic (2020–2022) was estimated on the basis of projection of the time series, using the APC value of the immediately preceding period segment. It was assumed that, in the absence of the pandemic, mortality by CVD subgroup among the different groups would follow the same trend observed previously. Thus, the number of deaths recorded in 2019 was used as a reference, applying the trend coefficient (APC) to estimate expected deaths in subsequent years. Finally, the standardized mortality ratio (SMR) was calculated by dividing the observed values by those expected for each year from 2020 to 2022.

The research project was approved by the Research Ethics Committee of the Sérgio Arouca National School of Public Health, of the Oswaldo Cruz Foundation, under Approval No. 6.263.870.

## RESULTS

The analysis of CVD mortality rates in Brazil between 2000 and 2022 revealed distinct patterns of decline and stabilization, with significant variations between disease subgroups, sexes and different age groups (Table 1). IHD and CVD showed a predominantly decreasing trend throughout the period, while the group of other CVD showed more heterogeneous behavior, with trend reversals in more recent years. The year 2020 emerged as a milestone in the historical series, characterized by changes in mortality trends, possibly due to the direct and indirect impacts of the COVID-19 pandemic.

**Table 1:** Average annual percent change and annual percent change in mortality rate by cardiovascular disease subgroup according to sex and age group. Brazil, 2000–2022.

Variable	Category	IHD		CBVD		Other CVD	
		Period	APC (95%CI)	Segment	APC (95%CI)	Segment	APC (95%CI)
Sex	Males	2000–2017	-0.8 (-1.0 to −0.6)*	2000–2008	-1.5 (-2.0 to −0.9)*	2000–2002	-2.5 (-4.2–0.0)
		2017–2020	-4.3 (-5.2 to −0.5)*	2008–2020	-3.0 (-3.9 to −2.3)*	2002–2007	0.7 (-1.9–2.2)
		2020–2022	0.4 (-2.7–2.3)	2020–2022	-0.2 (-2.8–1.1)	2007–2020	-1.7 (-3.5 to −1.1)*
						2020–2022	3.8 (0.3–5.7)*
		**AAPC (95%CI)**	-1.2 (-1.3 to −1.0)*	**AAPC (95%CI)**	-2.2 (-2.4 to −2.1)*	**AAPC (95%CI)**	-0.7 (-0.9 to −0.5)*
	Females	2000–2017	-1.2 (-1.4 to −1.1)*	2000–2007	-1.3 (-2.0 to −0.4)*	2000–2002	-3.0 (-5.1–0.0)
		2017–2020	-5.5 (-6.5 to −3.6)*	2007–2016	-3.0 (-3.2 to −1.1)*	2002–2007	0.4 (-2.8–2.7)
		2020–2022	0.6 (-2.1–2.7)	2016–2020	-4.4 (-5.8 to −3.4)*	2007–2020	-1.7 (-3.5 to −1.1)*
				2020–2022	0.4 (-2.2–2.3)	2020–2022	3.8 (0.3–5.7)*
		**AAPC (95%CI)**	-1.7 (-1.8 to −1.5)*	**AAPC (95%CI)**	-2.4 (-2.6 to −2.3)*	**AAPC (95%CI)**	-0.9 (-1.1 to −0.6)*
Age group (years)	0–19	2000–2014	8.3 (6.0–20.7)*	2000–2022	-0.1 (-0.7–0.5)	2000–2016	-1.3 (-1.8–0.3)
		2014–2022	-1.0 (-15.9–4.1)			2016–2022	-4.4 (-9.8 to −2.4)*
		**AAPC (95%CI)**	4.8 (2.6–7.1)*	**AAPC (95%CI)**	-0.1 (-0.7–0.5)	**AAPC (95%CI)**	-2.2 (-2.7 to −1.6)*
	20–29	2000–2002	-4.6 (-7.4–0.6)	2000–2004	-5.2 (-10.3–0.3)	2000–2020	-1.4 (-2.1 to −1.1)*
		2002–2012	2.3 (1.4–3.3)*	2004–2018	-1.4 (-2.4 to −0.5)*	2020–2022	4.6 (-1.0–7.6)
		2012–2015	9.7 (6.4–11.7)*	2018–2022	2.1 (-3.4–8.0)		
		2015–2018	-4.6 (-6.6 to −1.4)*				
		2018–2022	5.0 (3.2–9.5)*				
		**AAPC (95%CI)**	2.1 (1.8–2.6)*	**AAPC (95%CI)**	-1.5 (-2.9 to −0.1)*	**AAPC (95%CI)**	-0.8 (-1.3 to −0.6)*
	30–39	2000–2005	-3.0 (-4.3 to −1.6)*	2000–2009	-4.3 (-5.7 to −3.7)*	2000–2020	-1.5 (-1.8 to −1.3)*
		2005–2008	1.6 (-4.6–8.2)	2009–2020	-2.7 (-4.8 to −2.2)*	2020–2022	1.6 (-7.2–11.3)
		2008–2016	-0.2 (-1.0–0.7)	2020–2022	1.4 (-2.3–4.3)		
		2016–2019	-3.7 (-9.6–2.5)				
		2019–2022	3.6 (0.4–6.9)*				
		**AAPC (95%CI)**	-0.6 (-1.7–0.6)	**AAPC (95%CI)**	-3.0 (-3.3 to −2.8)*	**AAPC (95%CI)**	-1.3 (-2.0 to −0.5)*
**Variable**	**Category**	**IHD**		**CBVD**		**Other CVD**	
		**Period**	**APC (95%CI)**	**Segment**	**APC (95%CI)**	**Segment**	**APC (95%CI)**
Age group (years)	40–49	2000–2005	-2.8 (-5.4 to −1.8)*	2000–2020	-3.9 (-4.2 to −3.7)*	2000–2020	-1.8 (-2.2 to −1.7)*
		2005–2016	-1.1 (-1.4–0.7)	2020–2022	4.7 (-1.7–7.4)	2020–2022	5.1 (-0.3–7.5)
		2016–2020	-4.5 (-6.8 to −3.2)*				
		2020–2022	3.8 (0.2–6.5)*				
		**AAPC (95%CI)**	-1.7 (-1.9 to −1.5)*	**AAPC (95%CI)**	-3.2 (-3.6 to −2.9)*	**AAPC (95%CI)**	-1.2 (-1.6 to −1.0)*
	50–59	2000–2017	-1.4 (-2.5 to −0.9)*	2000–2019	-3.7 (-3.9 to −3.5)*	2000–2002	-4.1 (-5.8 to −1.5)*
		2017–2020	-5.2 (-6.9–0.2)	2019–2022	0.5 (-1.6–3.6)	2002–2008	-0.8 (-1.9–0.7)
		2020–2022	1.8 (-3.3–5.0)			2008–2020	-2.2 (-2.7 to −2.0)*
						2020–2022	5.0 (2.2–6.8)*
		**AAPC (95%CI)**	-1.6 (-2.0 to −1.4)*	**AAPC (95%CI)**	-3.1 (-3.3 to −3.0)*	**AAPC (95%CI)**	-1.4 (-1.6 to −1.2)*
	60–69	2000–2017	-1.3 (-1.4 to −1.1)*	2000–2020	-3.1 (-3.5 to −3.0)*	2000–2020	-1.8 (-2.5 to −1.5)*
		2017–2020	-4.1 (-8.2–0.3)	2020–2022	-0.3 (-3.0–1.1)	2020–2022	3.8 (-1.6–6.4)
		2020–2022	-0.0 (-4.3–4.5)				
		**AAPC (95%CI)**	-1.6 (-2.2 to −0.9)*	**AAPC (95%CI)**	-2.8 (-3.1 to −2.7)*	**AAPC (95%CI)**	-1.3 (-1.7 to −1.1)*
	70 and over	2000–2017	-0.7 (-0.8 to −0.5)*	2000–2008	-0.1 (-0.5–0.5)	2000–2008	0.8 (0.1–1.6)*
		2017–2020	-5.3 (-8.7 to −1.7)*	2008–2016	-2.7 (-3.1 to −1.2)*	2008–2020	-1.4 (-3.2 to −1.1)*
		2020–2022	-0.2 (-3.8–3.6)	2016–2020	-4.5 (-5.8 to −3.4)*	2020–2022	3.8 (-0.6–5.8)
				2020–2022	-0.1 (-2.4–1.8)		
		**AAPC (95%CI)**	-1.3 (-1.8 to −0.7)*	**AAPC (95%CI)**	-1.8 (-2.0 to −1.7)*	**AAPC (95%CI)**	-0.2 (-0.5–0.0)
**Total** ^a^		2000–2017	-1,0 (-1,2 to −0,8)*	2000–2008	-1.5 (-1.8 to −1.0)*	2000–2002	-2.8 (-4.3 to −0.1)*
		2017–2020	-4,8 (-5,9 to −0,7)*	2008–2020	-3.2 (-3.8 to −3.0)*	2002–2007	0.5 (-2.0–2.2)
		2020–2022	0,6 (-2,9–2,7)	2020–2022	-0.4 (-2.7–0.9)	2007–2020	-1.7 (-2.1 to −1.4)*
		**AAPC (95%CI)**	**-1,4 (-1,6 to −1,2)** *	**AAPC (95%CI)**	**-2.3 (-2.5 to −2.2)** *	**2020–2022**	**4.1 (1.6–5.8)** *
						**AAPC (95%CI)**	**-0.8 (-0.9 to −0.6)** *

IHD: ischemic heart disease; CBVD: cerebrovascular disease; CVD: cardiovascular disease; APC: annual percent change; AAPC: average annual percent change; 95%CI: 95% confidence interval;

* p<0.05; rate standardized by sex and age group

In the IHD subgroup, a sustained reduction in mortality was observed until 2017, with average annual declines of −0.8% for men and −1.2% for women. This decline was even more pronounced in the period from 2017 to 2020, reaching −4.3% per year in men and −5.5% in women, possibly reflecting advances in the prevention and management of these diseases. However, in 2020, there was a slowdown in this trend, with values that, although statistically not significant, suggested a possible stabilization (men: +0.4%; women: +0.6%).

Regarding CVD, mortality showed a significant and sustained reduction over the period from 2000 to 2020, with annual decline rates ranging from −3 to −3.2%. However, unlike IHD, there was no reversal of the trend after 2020, but rather a slowdown in the decline, with variations of −0.2% for men and +0.4% for women. The magnitude of AAPC confirms this pattern, being −2.2% for men and −2.4% for women, which suggests that this subgroup showed the most significant decline over the period analyzed.

In contrast, mortality due to other CVD followed a different behavior from the other subgroups. Initially, there was an oscillation without a clear trend until 2007, followed by a moderate reduction until 2020. However, in that year, there was a notable reversal, with a significant increase in mortality in both sexes (+3.8% per year). This phenomenon suggests that the aforementioned subgroup may have been the most impacted by the changes that occurred during the pandemic, possibly reflecting factors such as delays in diagnosis and treatment of less common but potentially lethal cardiovascular conditions.

When analyzing the differences between the sexes, it was found that women presented more pronounced reductions in mortality from IHD (-1.7% per year, compared with −1.2% in men), while for CVD the difference was smaller (-2.4% versus −2.2%). In the subgroup of other CVD, the discrepancy was even less pronounced (-0.9% for women versus −0.7% for men). These results indicate that the reduction in mortality from CVD was more significant among women, especially in the IHD subgroup, where the percent difference between the sexes reached 29.2%.

The analysis by age group also revealed different patterns. In the IHD subgroup, the population aged 0–19 years showed an increase in mortality between 2000 and 2014 (+8.3%), followed by a reversal of the trend with a decrease of −1% between 2014 and 2022. The largest reductions occurred in the age groups of 40–49 years (-3.2% per year) and 50–59 years (-3.1%); however, these same age groups registered a reversal of the trend after 2020, suggesting a possible impact of the pandemic. Additionally, the group aged 20–29 years showed the largest fluctuations over the period, alternating between sharp growth (+9.7% between 2012 and 2015) and significant reductions (-4.6% from 2015 to 2018), ending with a new substantial increase (+5% between 2018 and 2022). These variations may reflect changes in risk factors and health conditions of this population over time.

The year 2020 stood out as a turning point for cardiovascular mortality trends in Brazil. It was observed that while the reduction in mortality due to IHD did not show a significant upward trend and that the trend for CBVD was slowed, the subgroup of other CVD showed a significant increase in mortality, configuring itself as the only subgroup to reverse the downward trend observed until then. Regarding sex, both the IHD and CBVD groups did not show a significant trend for the 2020–2022 triennium, only for other CVD. In addition, intermediate age groups, such as 40–49 years for IHD and 50–59 years for other CVD, which showed consistent reductions in previous decades, registered an increase in the period 2020–2022, reinforcing the hypothesis that the pandemic had a significant influence on cardiovascular mortality patterns selectively to certain population groups.

The results in Table 2, on excess mortality by CVD subgroups during the COVID-19 pandemic period between 2020 and 2022, show an increasing pattern of excess mortality by CVD subgroups during the years of the COVID-19 pandemic (2020–2022). In 2020, an initial increase of 4% was observed in the other CVD subgroup (SMR=1.04; 95%CI 1.03–1.05), while CBVD and IHD remained stable. However, in 2021, all subgroups registered a significant excess: CBVD and IHD showed an 8% increase (SMR=1.08; 95%CI 1.07–1.09), and other CVD reached 15% (SMR=1.15; 95%CI 1.14–1.16). This pattern intensified in 2022, with rates of 16% for CBVD (SMR=1.16), 18% for IHD (SMR=1.18) and 23% for other CVD (SMR=1.23), reinforcing the upward trend over the period.

**Table 2:** Excess mortality by cardiovascular disease subgroup according to population group, 2020-2022.

Population group	2020			2021		
	CBVD	IHD	Other CVD	CBVD	IHD	Other CVD
	SMR (95%CI)	SMR (95%CI)	SMR (95%CI)	SMR (95%CI)	SMR (95%CI)	SMR (95%CI)
Males	1.01 (1.00–1.02)	0.98 (0.97–0.99)	1.04 (1.03–1.05)	1.07 (1.06–1.08)	1.07 (1.06–1.08)	1.15 (1.14–1.16)
Females	1.01 (1.00–1.02)	0.96 (0.95–0.97)	1.04 (1.03–1.05)	1.11 (1.10–1.12)	1.09 (1.08–1.10)	1.16 (1.15–1.17)
0 to 19 years	0.87 (0.77–0.97)	1.09 (0.92–1.27)	0.88 (0.82–0.94)	0.91 (0.81–1.02)	1.18 (0.99–1.36)	0.89 (0.83–0.95)
20 to 29 years	0.96 (0.88–1.04)	1.09 (1.01–1.18)	0.95 (0.90–1.01)	1.16 (1.07–1.25)	1.15 (1.07–1.24)	1.04 (0.98–1.10)
30 to 39 years	1.03 (0.98–1.08)	1.03 (0.99–1.08)	0.97 (0.94–1.01)	1.09 (1.04–1.14)	1.17 (1.12–1.22)	1.04 (1.00–1.07)
40 to 49 years	0.98 (0.95–1.01)	0.99 (0.96–1.01)	0.98 (0.95–1.00)	1.11 (1.08–1.14)	1.06 (1.04–1.09)	1.09 (1.06–1.11)
50 to 59 years	1.01 (0.99–1.03)	0.94 (0.93–0.96)	1.02 (1.00–1.03)	1.08 (1.06–1.10)	1.01 (1.00–1.03)	1.11 (1.09–1.12)
60 to 69 years	0.99 (0.98–1.01)	0.93 (0.91–0.94)	1.01 (0.99–1.02)	1.03 (1.01–1.04)	0.99 (0.98–1.00)	1.10 (1.08–1.11)
70 and over	0.98 (0.97–0.99)	0.96 (0.95–0.97)	1.02 (1.01–1.03)	1.04 (1.03–1.05)	1.04 (1.03–1.05)	1.10 (1.09–1.11)
Total	1.00 (0.99–1.01)	0.97 (0.96–0.98)	1.04 (1.03–1.05)	1.08 (1.07–1.09)	1.08 (1.07–1.09)	1.15 (1.14–1.16)
**Population group**	**2022**					
	**CBVD**		**IHD**		**Other CVD**	
	**SMR (95%CI)**		**SMR (95%CI)**		**SMR (95%CI)**	
Males	1.16 (1.15–1.17)		1.17 (1.16–1.18)		1.23 (1.22–1.24)	
Females	1.20 (1.19–1.21)		1.20 (1.19–1.21)		1.23 (1.22–1.24)	
0 to 19 years	1.06 (0.95–1.17)		1.15 (0.96–1.34)		1.09 (1.02–1.16)	
20 to 29 years	1.07 (0.99–1.16)		1.35 (1.25–1.45)		1.13 (1.07–1.19)	
30 to 39 years	1.14 (1.08–1.19)		1.22 (1.17–1.28)		1.11 (1.08–1.15)	
40 to 49 years	1.14 (1.11–1.17)		1.19 (1.16–1.21)		1.15 (1.13–1.18)	
50 to 59 years	1.12 (1.10–1.14)		1.11 (1.10–1.13)		1.14 (1.13–1.16)	
60 to 69 years	1.07 (1.06–1.09)		1.06 (1.04–1.07)		1.13 (1.11–1.14)	
70 and over	1.11 (1.10–1.12)		1.11 (1.10–1.12)		1.14 (1.13–1.15)	
Total	1.16 (1.15–1.17)		1.18 (1.17–1.19)		1.23 (1.22–1.24)	

CBVD: cerebrovascular diseases; IHD: ischemic heart diseases; CVD: cardiovascular diseases; SMR: standardized mortality ratio; 95%CI: 95% confidence interval.

The analysis stratified by sex revealed that women had slightly higher excesses than men in 2021 (SMR=1.11 versus 1.07 for CBVD; 1.09 versus 1.07 for IHD) and 2022 (SMR=1.20 versus 1.16 for CBVD; 1.20 versus 1.17 for IHD).

Regarding age group, specific groups stood out by CVD subgroup that had an increasing pattern of excess mortality. In 2022, the greatest impact for the CBVD subgroup was between 30–39 and 40–49 years (both age groups with SMR=1.14) and 50–59 years (SMR=1.12). For IHD, there was a peak in adults aged 20–29 (SMR=1.35), 30–39 years (SMR=1.22) and 40–49 years (SMR=1.19). Regarding the other CVD subgroup, individuals ≥40 years were the most affected with SMR, which ranged 1.13–1.1.

## DISCUSSION

The results showed a general downward trend in CVD mortality in Brazil between 2000 and 2020, with significant differences between subgroups, sexes and age groups. However, in 2020, there was a change in these trajectories, characterized by a slowdown in the decline and, in some cases, a reversal of the trend.

Before the pandemic, Brazil had recorded a continuous decline in age-adjusted mortality rates for CVD, but recent studies indicated that this trend was interrupted or even reversed during the pandemic^8^. Data from the Mortality Information System revealed an 8.8% reduction in CVD deaths between March and December 2020, compared to the same period in 2019. This decrease can be attributed to underreporting or changes in death registration patterns during the health crisis^8^. Furthermore, a significant increase in deaths due to CVD as a comorbidity was observed in patients with COVID-19, suggesting that infection by SARS-CoV-2 exacerbated preexisting cardiovascular conditions^9^.

The pandemic affected CVD subgroups differently. IHD, which includes conditions such as myocardial infarction, showed a reduction in hospitalizations during the critical periods of the pandemic. This decrease may have been a result of the population's fear of seeking medical care because of the risk of contamination, leading to delays in diagnosis and treatment and possibly increasing home mortality due to acute cardiac events^10^.

In the case of CBVD, such as stroke, there was also a reduction in hospital admissions. Studies have suggested that patients with mild symptoms may have avoided seeking immediate care, resulting in worsening clinical outcomes^10^. In addition, the overload of health systems may have limited access to emergency therapies, which are essential to minimize neurological sequelae^11^. The subgroup of other CVD, which includes valvular diseases and cardiomyopathies, faced additional challenges. Patients with advanced valvular conditions, for example, had surgical or interventional procedures postponed due to the prioritization of resources for combating COVID-19^11^. This postponement may have contributed to the progression of the disease and increased mortality in this specific group^11^.

Analysis by age group shows that older individuals were more vulnerable. Studies have shown that patients with CVD and COVID-19 tend to be older, with a higher average age than patients without CVD^12^. This greater susceptibility can be attributed to the presence of comorbidities and the immunological fragility associated with aging^12^. Regarding sex, although both sexes had been affected, some studies indicate that men with CVD may have higher rates of complications when infected with COVID-19, possibly because of hormonal and behavioral factors^13^.

The greater susceptibility of age groups over 40 years to trend reversals in CVD mortality during the pandemic can be explained by several factors, including the prevalence of comorbidities, the systemic effects of COVID-19 and the inflammatory and microvascular characteristics associated with both aging and SARS-CoV-2 infection itself^11,14^. In fact, advanced age is one of the main risk factors for CVD. Individuals over 40 years of age have a higher prevalence of conditions such as hypertension, diabetes mellitus, dyslipidemia and obesity, which are key determinants in the development and progression of CVD. During the pandemic, the presence of these comorbidities has been widely documented as an aggravating factor for the severity of COVID-19 and for the increased risk of adverse outcomes, including death from CVD^10,15^.

Furthermore, the impact of the pandemic on these individuals may have been exacerbated by the discontinuation of regular medical monitoring and the delay in the diagnosis and treatment of acute cardiovascular events, such as myocardial infarction and stroke^9^. This interruption may have contributed to the worsening of chronic disease control, increasing the incidence of fatal cardiovascular decompensations.

Furthermore, the inflammatory storm induced by COVID-19 also plays a critical role in worsening cardiovascular outcomes. The increase in pro-inflammatory cytokines, such as interleukin-6 (IL-6) and tumor necrosis factor-alpha (TNF-α), can trigger hypercoagulable states, contributing to thrombotic events, including infarctions and strokes^16^. This process may explain the increased mortality from CVD in older individuals, who are already more prone to thrombotic events because of advanced atherosclerosis and endothelial dysfunction.

Differences in cardiovascular mortality between the sexes during the pandemic may also be influenced by behavioral factors. Pre-pandemic studies already indicated that men tend to seek less preventive medical care and have lower adherence to treatment for chronic diseases, such as hypertension and diabetes, when compared to women^17^. During the pandemic, this trend may have been exacerbated by fear of contamination in health services, leading to a higher number of cardiovascular decompensations and fatal events at home.

In contrast, some evidence suggests that women were more impacted by the interruption of non-emergency medical services during the pandemic, especially in relation to follow-up consultations and preventive examinations^18^. This factor may have particularly affected women with previous CVD, increasing the risk of adverse outcomes in the medium term.

In addition, differences in the socioeconomic impact of the pandemic may have affected men and women differently. Household overload and inequality in access to health services during the pandemic may have resulted in differential outcomes between the sexes, reinforcing the need for personalized approaches to mitigate the cardiovascular impact of COVID-19^19^.

The excess mortality observed in this study, mainly in 2021 and 2022, corroborates the findings of the time series analysis, with significant magnitudes that suggest a phenomenon of anticipation of deaths (harvesting effect). This pattern indicates that deaths that would likely occur in a longer time horizon were precipitated by factors interconnected with the pandemic context, such as restricted access to health services — including slowdown in treatments, hospitalizations and regular follow-ups —, in addition to the worsening of comorbidities due to the interruption of chronic care. Structural inequalities in access to health, intensified during the crisis, were reflected in the increase in in-hospital mortality due to CVD^20^ and in the reduction of elective hospitalizations^9,21^, a scenario associated with the greater occurrence of home deaths^22^ and the discontinuity of care for high-risk patients^23^.

Additionally, social isolation contributed to the increase in modifiable risk factors, such as sedentary lifestyle and stress, increasing cardiovascular vulnerability^24^. In 2020, there was a 22% excess mortality rate due to natural causes in Brazil, with CVD accounting for a significant portion of this increase^25^. The CVD group showed an excess of deaths of 16%^26^, reaching 77.9% when considered as a comorbidity of deaths^8^. Despite possible limitations in the quality of mortality records during the pandemic, the consistency of the data reinforces the substantial influence of the health crisis on cardiovascular morbidity and mortality, evidencing not only the direct burden of SARS-CoV-2 infection, but also the indirect effects of the disorganization of health systems and behavioral changes.

Ultimately, the data presented corroborate the hypothesis that the COVID-19 pandemic acted as a turning point in CVD mortality trends in Brazil, affecting various age groups and both sexes. The interruption of previous declining trends, associated with the increase in deaths from CVD as a comorbidity in patients with COVID-19, highlights the significant impact of the pandemic on the cardiovascular health of the population^8,9^.

Furthermore, the differences observed between CVD subgroups reinforce the second part of the hypothesis. While IHD and CBVD showed reductions in hospitalizations possibly related to fear of contamination and overload of health services^12,13^, other CVD, such as valve diseases, suffered from the postponement of essential procedures, leading to adverse outcomes^13^.

In summary, the methods employed, based on time series analysis and joinpoint regression, allowed us to identify significant changes in CVD mortality trends during the COVID-19 pandemic in Brazil. The slowdown in the decline of IHD and CBVD, as well as the abrupt increase in other CVD from 2020 onwards, suggests that the indirect impacts of the pandemic — such as reduced access to health services, postponement of elective procedures, and psychosocial stress — may have exacerbated preexisting vulnerabilities, especially in women and intermediate age groups (40–59 years). The methodology for projecting expected deaths, anchored in historical trends, reinforces the robustness of the estimate of excess mortality (up to 23% in 2022), in line with international studies^10,11^ that reported similar patterns of cardiovascular deterioration during health crises.

In terms of limitations, we acknowledge that the reliance on secondary data from the Mortality Information System may lead to diagnostic errors, underreporting of deaths, and inconsistencies in the coding of causes of death, factors that underestimate the true magnitude of CVD mortality. In addition, the lack of detailed information on comorbidities and differentiated regional access to health services limits the understanding of specific causal mechanisms. Despite these limitations, the results provide a coherent and relevant view of the dynamics of cardiovascular mortality during the pandemic, highlighting critical patterns such as the significant increase in other CVD and the vulnerability of intermediate age groups.

These findings reinforce the need for public policies that ensure continuity of care for chronic patients during crises, in addition to investments in more accurate surveillance systems to capture regional and diagnostic nuances. Stratification by sex and age, although subject to data limitations, highlights disparities that require targeted interventions, consolidating the study as a crucial empirical basis for guiding mitigation strategies in future health emergency scenarios.
