# Supplementary material for: Analysis of mortality by cardiovascular disease subgroups in Brazil before and during the COVID-19 pandemic (2000–2022) by sex and age group
Source: Rev Bras Epidemiol. 2025 Jun 27;28:e250033. doi: 10.1590/1980-549720250033 (PMC12204235; doi:10.1590/1980-549720250033)
Supplement: Translation

# Translation: Análise da mortalidade por subgrupos de doenças cardiovasculares no Brasil antes e ao longo da pandemia por COVID-19 (2000–2022) segundo sexo e grupos etários

**DOI:** 10.1590/1980-549720250033.2

**Authors:** Cicero Emanoel Alves Leite, Raphael Mendonça Guimarães, Andréa Sobral

**Affiliations:** I Fundação Oswaldo Cruz, Escola Nacional de Saúde Pública Sérgio Arouca, Programa de Pós-Graduação em Saúde Pública e Meio Ambiente – Rio de Janeiro (RJ), Brasil.; II Fundação Oswaldo Cruz, Escola Nacional de Saúde Pública Sérgio Arouca, Departamento de Ciências Sociais – Rio de Janeiro (RJ), Brasil.; III Fundação Oswaldo Cruz, Escola Nacional de Saúde Pública Sérgio Arouca, Departamento de Endemias Samuel Pessoa – Rio de Janeiro (RJ), Brasil.

**Keywords:** Estudos de séries temporais, Comorbidade, Doenças não transmissíveis, COVID-19, Mortalidade

## Abstract

**Objetivo:**

Analisar o efeito da pandemia de COVID-19 na mortalidade por subgrupos de doenças cardiovasculares (DCV) no Brasil, considerando sexo e grupo etário.

**Métodos:**

Estudo ecológico de séries temporais com dados secundários do Sistema de Informações sobre Mortalidade referentes ao período 2000–2022. Calcularam-se taxas de mortalidade ajustadas pela idade por 100 mil habitante, e utilizaram-se modelos de regressão por *joinpoint* para estimar as tendências das taxas.

**Resultados:**

A mortalidade por DCV no Brasil entre 2000 e 2022 mostrou queda nas doenças isquêmicas do coração (DIC) e cerebrovasculares (DCbV), enquanto outras DCV tiveram comportamento instável, com aumento após 2020. A redução das DIC foi mais acentuada até 2020, desacelerando posteriormente. As DCbV mantiveram queda, mas em ritmo menor. Já outras DCV, após tendência de redução, apresentaram alta significativa (+3,8% ao ano), possivelmente por causa da pandemia. As mulheres tiveram maior redução da mortalidade, especialmente por DIC. Faixas etárias intermediárias (40–59 anos) mostraram queda até 2020, mas registraram aumento após esse período, reforçando o impacto da pandemia. Em 2021, todos os subgrupos apresentaram excesso de mortalidade: 8% para DCbV e DIC e 15% para outras DCV. Em 2022, esse padrão intensificou-se, com valores de 16% (DCbV), 18% (DIC) e 23% (outras DCV).

**Conclusão:**

A pandemia de COVID-19 impactou significativamente a mortalidade por DCV no Brasil, alterando tendências conforme faixa etária, sexo e subgrupo de doença. O estudo aponta para uma inflexão nas taxas de mortalidade durante a pandemia, destacando a necessidade de investigações adicionais para compreender os mecanismos envolvidos.

## INTRODUÇÃO

As doenças cardiovasculares (DCV) são responsáveis por cerca de 17,9 milhões de óbitos por ano, a principal causa de mortalidade em todo o mundo^1^ e estão associadas a fatores sociodemográficos, de saúde e ao estilo de vida^2^. No Brasil em 2019, as doenças isquêmicas do coração e doenças cerebrovasculares foram responsáveis por 21,4% do total de óbitos no geral^3^. Em 2021, a prevalência de DCV foi de 6,9% na população brasileira, sendo mais alta entre homens (7,6%) do que mulheres (6,3%)^4^. Além do efeito na mortalidade, esse grupo de doenças reduz a expectativa de vida e causa incapacidades^5^.

Todavia, eventos de grande impacto na saúde pública, como a pandemia de COVID-19, podem modificar essas tendências, seja por efeitos diretos da infecção pelo Sars-CoV-2, seja por impactos indiretos, como restrições ao acesso a serviços de saúde e mudanças no comportamento da população.

As doenças infecciosas e parasitárias passaram a ser a principal causa de morte, por causa da alta carga de COVID-19 em 2021^5^. Pessoas com comorbidades, incluindo as DCV, exerceram forte pressão no sistema de saúde por apresentarem risco aumentado para óbito em razão da COVID-19^6^. No Brasil, os serviços de saúde foram reorganizados durante a pandemia para mitigar o risco de contágio e garantir assistência aos pacientes com COVID-19, o que afetou o atendimento de pessoas com comorbidades^7^.

Nesse contexto, compreender como a pandemia influenciou a mortalidade por diferentes subgrupos de DCV, estratificados por sexo e grupo etário, é essencial para avaliar possíveis retrocessos nos avanços obtidos nas últimas décadas e para orientar políticas públicas de mitigação. Assim, este estudo investigou o efeito da pandemia de COVID-19 na mortalidade por subgrupos de DCV no Brasil, considerando sexo e grupo etário entre 2000 e 2022.

## MÉTODOS

Este estudo é uma análise de séries temporais, abordando as taxas de mortalidade em subgrupos de DCV ao longo do período de 2000 a 2022. Para essa análise, foram definidos, com base na 10ª edição da Classificação Internacional de Doenças e Problemas Relacionados à Saúde (CID-10), os subgrupos de DCV: doenças isquêmicas do coração (DIC), doenças cerebrovasculares (DCbV) e outras DCV (Quadro 1).

**Quadro 1:** Categoria da 10ª edição da Classificação Internacional de Doenças e Problemas Relacionados à Saúde (CID-10) de acordo com o subgrupo de doenças cardiovasculares (DCV).

Subgrupo de DCV	Categoria CID-10
Doenças isquêmicas do coração (DIC)	I20 *Angina pectoris*
	I21 Infarto agudo do miocárdio
	I22 Infarto do miocárdio recorrente
	I23 Algumas complicações atuais subsequentes ao infarto agudo do miocárdio
	I24 Outras doenças isquêmicas agudas do coração
	I25 Doença isquêmica crônica do coração
Doenças cerebrovasculares (DCbV)	I60 Hemorragia subaracnoidea
	I61 Hemorragia intracerebral
	I62 Outras hemorragias intracranianas não traumáticas
	I63 Infarto cerebral
	I64 Acidente vascular cerebral não especificado como hemorragia isquêmica
	I65 Oclusão e estenose de artérias pré-cerebrais que não resultam em infarto cerebral
	I66 Oclusão e estenose de artérias cerebrais que não resultam em infarto cerebral
	I67 Outras doenças cerebrovasculares
	I68 Transtornos cerebrovasculares em doenças classificadas em outra parte
	I69 Sequelas de doenças cerebrovasculares
Outras DCV	I00 Febre reumática sem menção de comprometimento do coração
	I01 Febre reumática com comprometimento do coração
	I02 Coreia reumática
	I05 Doenças reumáticas da valva mitral
	I06 Doenças reumáticas da valva aórtica
	I07 Doenças reumáticas da valva tricúspide
	I08 Doenças de múltiplas valvas
	I09 Outras doenças reumáticas do coração
	I10 Hipertensão essencial
Outras DCV	I11 Doença cardíaca hipertensiva
	I12 Doença renal hipertensiva
	I13 Doença cardíaca e renal hipertensiva
	I15 Hipertensão secundária
	I26 Embolia pulmonar
	I27 Outra forma de doença cardíaca pulmonar
	I28 Outra doença dos vasos pulmonares
	I30 Pericardite aguda
	I31 Outra doença do pericárdio
	I32 Pericardite em doenças classificadas em outra parte
	I33 Endocardite aguda e subaguda
	I34 Transtornos não reumáticos da valva mitral
	I35 Transtornos não reumáticos da valva aórtica
	I36 Transtornos não reumáticos da valva tricúspide
	I37 Transtornos da valva pulmonar
	I38 Endocardite de valva não especificado
	I39 Endocardite e transtornos valvulares cardíacos em doenças classificadas em outra parte
	I40 Miocardite aguda
	I41 Miocardite em doenças classificadas em outra parte
	I42 Cardiomiopatias
	I43 Cardiomiopatia em doenças classificadas em outra parte
	I44 Bloqueio atrioventricular e do ramo esquerdo
	I45 Outros transtornos de condução
	I46 Parada cardíaca
	I47 Taquicardia paroxística
	I48 Flutter e fibrilação atrial
	I49 Outras arritmias cardíacas
	I50 Insuficiência cardíaca
	I51 Complicações de cardiopatias e doenças cardíacas mal definidas
	I52 Outras afecções cardíacas em doenças classificadas em outra parte
	I70 Aterosclerose
	I71 Aneurisma e dissecção da aorta
	I72 Outros aneurismas
	I73 Outras doenças vasculares periféricas
	I74 Embolia e trombose arteriais
	I77 Outras afecções das artérias e arteríolas
	I78 Doença dos capilares
	I79 Transtorno das artérias, das arteríolas e dos capilares em doenças classificadas em outra parte
	I80 Flebite e tromboflebite
	I81 Trombose da veia porta
	I82 Outra embolia e trombose venosas
	I83 Varizes dos membros inferiores
	I84 Hemorroidas
	I85 Varizes esofagianas
	I86 Varizes de outras localizações
	I87 Outros transtornos das veias
	I88 Linfadenite inespecífica
	I89 Outros transtornos não-infecciosos dos vasos linfáticos e dos gânglios linfáticos
	I95 Hipotensão
	I97 Transtornos do aparelho circulatório subsequentes a procedimentos não classificados em outra parte
	I98 Outros transtornos do aparelho circulatório em doenças classificadas em outra parte
	I99 Outros transtornos do aparelho circulatório e os não especificados

Os dados foram obtidos do Sistema de Informações sobre Mortalidade, disponibilizados publicamente pelo Departamento de Informática do Sistema Único de Saúde, e categorizados pelos subgrupos de DCV diferenciando as variáveis sexo, ano do óbito e idade categorizada por faixa etária. A base populacional utilizada foi a das projeções e retroprojeções realizadas pelo Instituto Brasileiro de Geografia e Estatística para cada localidade e ano.

Para analisar as tendências, realizamos a regressão segmentada, por meio do programa Joinpoint Regression 5.0, que identifica inflexões na tendência temporal ao longo do tempo. Foram calculadas taxas anuais ajustadas pela idade, assumindo variância constante (homocedasticidade). O modelo foi selecionado usando o *weighted Bayesian information criterion*. Além disso, foram empregados intervalos de confiança (IC) para estimar a variação percentual anual (VPA) e a variação percentual anual média (VPAM) e determinar pontos de mudança com o método quantil empírico. As tendências (VPA e VPAM) foram classificadas como estável (p³0,05 e coeficiente da regressão positivo ou negativo), crescente (p<0,05 e coeficiente positivo) e decrescente (p<0,05 e coeficiente negativo).

Para a análise do excesso de mortalidade de cada grupo populacional, estimou-se a quantidade de óbitos esperada para os anos da pandemia de COVID-19 (2020–2022) com base na projeção da série temporal, utilizando o valor do VPA do segmento de período imediatamente anterior. Partiu-se do pressuposto de que, na ausência da pandemia, a mortalidade por subgrupo de DCV entre os diferentes grupos seguiria a mesma tendência observada previamente. Dessa forma, o número de óbitos registrados em 2019 foi usado como referência, aplicando-se o coeficiente da tendência (VPA) para estimar os óbitos esperados nos anos subsequentes. Por fim, calculou-se a razão de mortalidade padronizada (SMR) dividindo os valores observados pelos esperados para cada ano do período de 2020 a 2022.

O projeto de pesquisa foi aprovado pelo Comitê de Ética em Pesquisa da Escola Nacional de Saúde Pública Sérgio Arouca, da Fundação Oswaldo Cruz, com o Parecer nº 6.263.870.

## RESULTADOS

A análise das taxas de mortalidade por DCV no Brasil entre 2000 e 2022 revelou padrões distintos de declínio e estabilização, com variações significativas entre os subgrupos de doenças, os sexos e as diferentes faixas etárias (Tabela 1). As DIC e as DCbV apresentaram tendência predominantemente decrescente ao longo do período, enquanto o grupo de outras DCV demonstrou comportamento mais heterogêneo, com inversões de tendência nos anos mais recentes. O ano de 2020 emergiu como um marco na série histórica, caracterizando-se por alterações nas tendências de mortalidade, possivelmente em decorrência dos impactos diretos e indiretos da pandemia de COVID-19.

**Tabela 1:** Variação percentual anual média e variação percentual anual da taxa de mortalidade por subgrupos de doenças cardiovasculares segundo sexo e faixa etária. Brasil, 2000–2022.

Variável	Categoria	DIC		DCbV		Outras DCV	
		Período	VPA (IC95%)	Segmento	VPA (IC95%)	Segmento	VPA (IC95%)
Sexo	Masculino	2000–2017	-0,8 (-1,0 a −0,6)*	2000–2008	-1,5 (-2,0 a −0,9)*	2000–2002	-2,5 (-4,2–0,0)
		2017–2020	-4,3 (-5,2 a −0,5)*	2008–2020	-3,0 (-3,9 a −2,3)*	2002–2007	0,7 (-1,9–2,2)
		2020–2022	0,4 (-2,7; 2,3)	2020–2022	-0,2 (-2,8–1,1)	2007–2020	-1,7 (-3,5 a −1,1)*
						2020–2022	3,8 (0,3–5,7)*
		**VPAM (IC95%)**	-1,2 (-1,3 a −1,0)*	**VPAM (IC95%)**	-2,2 (-2,4 a −2,1)*	**VPAM (IC95%)**	-0,7 (-0,9 a −0,5)*
	Feminino	2000–2017	-1,2 (-1,4 a −1,1)*	2000–2007	-1,3 (-2,0 a −0,4)*	2000–2002	-3,0 (-5,1–0,0)
		2017–2020	-5,5 (-6,5 a −3,6)*	2007–2016	-3,0 (-3,2 a −1,1)*	2002–2007	0,4 (-2,8–2,7)
		2020–2022	0,6 (-2,1–2,7)	2016–2020	-4,4 (-5,8 a −3,4)*	2007–2020	-1,7 (-3,5 a −1,1)*
				2020–2022	0,4 (-2,2–2,3)	2020–2022	3,8 (0,3–5,7)*
		**VPAM (IC 95%)**	-1,7 (-1,8 a −1,5)*	**VPAM (IC95%)**	-2,4 (-2,6 a −2,3)*	**VPAM (IC95%)**	-0,9 (-1,1 a −0,6)*
Grupo etário (anos)	0–19	2000–2014	8,3 (6,0–20,7)*	2000–2022	-0,1 (-0,7–0,5)	2000–2016	-1,3 (-1,8–0,3)
		2014–2022	-1,0 (-15,9–4,1)			2016–2022	-4,4 (-9,8 a −2,4)*
		**VPAM (IC95%)**	4,8 (2,6–7,1)*	**VPAM (IC95%)**	-0,1 (-0,7–0,5)	**VPAM (IC95%)**	-2,2 (-2,7 a −1,6)*
	20–29	2000–2002	-4,6 (-7,4–0,6)	2000–2004	-5,2 (-10,3–0,3)	2000–2020	-1,4 (-2,1 a −1,1)*
		2002–2012	2,3 (1,4–3,3)*	2004–2018	-1,4 (-2,4 a −0,5)*	2020–2022	4,6 (-1,0–7,6)
		2012–2015	9,7 (6,4–11,7)*	2018–2022	2,1 (-3,4–8,0)		
		2015–2018	-4,6 (-6,6 a −1,4)*				
		2018–2022	5,0 (3,2–9,5)*				
		**VPAM (IC95%)**	2,1 (1,8–2,6)*	**VPAM (IC95%)**	-1,5 (-2,9 a −0,1)*	**VPAM (IC95%)**	-0,8 (-1,3 a −0,6)*
	30–39	2000–2005	-3,0 (-4,3 a −1,6)*	2000–2009	-4,3 (-5,7 a −3,7)*	2000–2020	-1,5 (-1,8 a −1,3)*
		2005–2008	1,6 (-4,6–8,2)	2009–2020	-2,7 (-4,8 a −2,2)*	2020–2022	1,6 (-7,2–11,3)
		2008–2016	-0,2 (-1,0–0,7)	2020–2022	1,4 (-2,3–4,3)		
		2016–2019	-3,7 (-9,6–2,5)				
		2019–2022	3,6 (0,4–6,9)*				
		**VPAM (IC95%)**	-0,6 (-1,7–0,6)	**VPAM (IC95%)**	-3,0 (-3,3 a −2,8)*	**VPAM (IC95%)**	-1,3 (-2,0 a −0,5)*
**Variável**	**Categoria**	**DIC**		**DCbV**		**Outras DCV**	
		**Período**	**VPA (IC95%)**	**Segmento**	**VPA (IC95%)**	**Segmento**	**VPA (IC95%)**
Grupo etário (anos)	40–49	2000–2005	-2,8 (-5,4 a −1,8)*	2000–2020	-3,9 (-4,2 a −3,7)*	2000–2020	-1,8 (-2,2 a −1,7)*
		2005–2016	-1,1 (-1,4–0,7)	2020–2022	4,7 (-1,7–7,4)	2020–2022	5,1 (-0,3–7,5)
		2016–2020	-4,5 (-6,8 a −3,2)*				
		2020–2022	3,8 (0,2–6,5)*				
		**VPAM (IC95%)**	-1,7 (-1,9 a −1,5)*	**VPAM (IC95%)**	-3,2 (-3,6 a −2,9)*	**VPAM (IC95%)**	-1,2 (-1,6 a −1,0)*
	50–59	2000–2017	-1,4 (-2,5 a −0,9)*	2000–2019	-3,7 (-3,9 a −3,5)*	2000–2002	-4,1 (-5,8 a −1,5)*
		2017–2020	-5,2 (-6,9–0,2)	2019–2022	0,5 (-1,6–3,6)	2002–2008	-0,8 (-1,9–0,7)
		2020–2022	1,8 (-3,3–5,0)			2008–2020	-2,2 (-2,7 a −2,0)*
						2020–2022	5,0 (2,2–6,8)*
		**VPAM (IC95%)**	-1,6 (-2,0 a −1,4)*	**VPAM (IC95%)**	-3,1 (-3,3 a −3,0)*	**VPAM (IC95%)**	-1,4 (-1,6 a −1,2)*
	60–69	2000–2017	-1,3 (-1,4 a −1,1)*	2000–2020	-3,1 (-3,5 a −3,0)*	2000–2020	-1,8 (-2,5 a −1,5)*
		2017–2020	-4,1 (-8,2–0,3)	2020–2022	-0,3 (-3,0–1,1)	2020–2022	3,8 (-1,6–6,4)
		2020–2022	-0,0 (-4,3–4,5)				
		**VPAM (IC95%)**	-1,6 (-2,2 a −0,9)*	**VPAM (IC95%)**	-2,8 (-3,1 a −2,7)*	**VPAM (IC95%)**	-1,3 (-1,7 a −1,1)*
	70 e mais	2000–2017	-0,7 (-0,8 a −0,5)*	2000–2008	-0,1 (-0,5–0,5)	2000–2008	0,8 (0,1–1,6)*
		2017–2020	-5,3 (-8,7 a −1,7)*	2008–2016	-2,7 (-3,1 a −1,2)*	2008–2020	-1,4 (-3,2 a −1,1)*
		2020–2022	-0,2 (-3,8–3,6)	2016–2020	-4,5 (-5,8 a −3,4)*	2020–2022	3,8 (-0,6–5,8)
				2020–2022	-0,1 (-2,4–1,8)		
		**VPAM (IC95%)**	-1,3 (-1,8 a −0,7)*	**VPAM (IC95%)**	-1,8 (-2,0 a −1,7)*	**VPAM (IC95%)**	-0,2 (-0,5–0,0)
**Total** ^a^		2000–2017	-1,0 (-1,2 a −0,8)*	2000–2008	-1,5 (-1,8 a −1,0)*	2000–2002	-2,8 (-4,3 a −0,1)*
		2017–2020	-4,8 (-5,9 a −0,7)*	2008–2020	-3,2 (-3,8 a −3,0)*	2002–2007	0,5 (-2,0–2,2)
		2020–2022	0,6 (-2,9–2,7)	2020–2022	-0,4 (-2,7–0,9)	2007–2020	-1,7 (-2,1 a −1,4)*
		**VPAM (IC95%)**	**-1,4 (-1,6 a −1,2)** *	**VPAM (IC95%)**	**-2,3 (-2,5 a −2,2)** *	**2020–2022**	**4,1 (1,6–5,8)** *
						**VPAM (IC95%)**	**-0,8 (-0,9 a −0,6)** *

DIC: doenças isquêmicas do coração; DCbV: doenças cerebrovasculares; DCV: doenças cardiovasculares; VPA: variação percentual anual; VPAM: variação percentual anual média; IC95%: intervalo de confiança de 95%;

* p<0,05; ataxa padronizada por sexo e faixa etária.

No subgrupo das DIC, observou-se redução sustentada da mortalidade até 2017, com quedas anuais médias de −0,8% para homens e −1,2% para mulheres. Esse declínio foi ainda mais acentuado no período de 2017 a 2020, atingindo −4,3% ao ano nos homens e −5,5% nas mulheres, possivelmente refletindo avanços na prevenção e no manejo dessas doenças. Entretanto, em 2020, verificou-se desaceleração dessa tendência, com valores que, embora estatisticamente não significativos, sugeriam uma possível estabilização (homens: +0,4%; mulheres: +0,6%).

No que se refere às DCbV, a mortalidade apresentou redução expressiva e sustentada ao longo do período de 2000 a 2020, com taxas anuais de queda variando de −3 a −3,2%. No entanto, diferentemente das DIC, não se constatou reversão da tendência após 2020, mas sim desaceleração do declínio, com variações de −0,2% para homens e +0,4% para mulheres. A magnitude da VPAM confirma esse padrão, sendo −2,2% para homens e −2,4% para mulheres, o que sugere que esse subgrupo apresentou o declínio mais expressivo ao longo do período analisado.

Em contrapartida, a mortalidade por outras DCV seguiu um comportamento distinto dos demais subgrupos. Inicialmente, observou-se oscilação sem tendência clara até 2007, seguida por redução moderada até 2020. Entretanto, nesse ano, houve uma inversão notável, com crescimento significativo da mortalidade em ambos os sexos (+3,8% ao ano). Esse fenômeno sugere que o referido subgrupo pode ter sido o mais impactado pelas mudanças ocorridas durante a pandemia, possivelmente refletindo fatores como atraso no diagnóstico e no tratamento de condições cardiovasculares menos comuns, porém potencialmente letais.

Ao analisar as diferenças entre os sexos, verificou-se que as mulheres apresentaram reduções mais acentuadas da mortalidade por DIC (-1,7% ao ano, em comparação com −1,2% nos homens), enquanto para DCbV a diferença foi menor (-2,4% *versus* −2,2%). No subgrupo de outras DCV, a discrepância foi ainda menos pronunciada (-0,9% para mulheres *versus* −0,7% para homens). Esses resultados indicam que a redução da mortalidade por DCV foi mais significativa entre as mulheres, especialmente no subgrupo de DIC, onde a diferença percentual entre os sexos atingiu 29,2%.

A análise por faixa etária também revelou padrões diferenciados. No subgrupo de DIC, a população de 0–19 anos apresentou aumento na mortalidade entre 2000 e 2014 (+8,3%), seguido por uma reversão da tendência com queda de −1% entre 2014 e 2022. As maiores reduções ocorreram nas faixas etárias de 40–49 anos (-3,2% ao ano) e 50–59 anos (-3,1%), contudo essas mesmas faixas etárias registraram inversão de tendência após 2020, sugerindo um possível impacto da pandemia. Adicionalmente, o grupo de 20–29 anos apresentou as maiores oscilações ao longo do período, alternando entre crescimento acentuado (+9,7% entre 2012 e 2015) e reduções expressivas (-4,6% de 2015 a 2018), finalizando com um novo aumento substancial (+5% entre 2018 e 2022). Essas variações podem refletir mudanças nos fatores de risco e nas condições de saúde dessa população ao longo do tempo.

O ano de 2020 se destacou como um ponto de inflexão para as tendências de mortalidade cardiovascular no Brasil. Observou-se que, enquanto a redução da mortalidade por DIC não apresentou tendência de aumento significativa, e a tendência para as DCbV foi desacelerada, o subgrupo de outras DCV apresentou aumento expressivo da mortalidade, configurando-se como o único subgrupo a reverter a tendência de declínio constatada até então. Com relação ao sexo, tanto o grupo de DIC quanto o de DCbV não apresentou tendência significativa para o triênio 2020–2022, apenas para as outras DCV. Além disso, faixas etárias intermediárias, como 40–49 anos para DIC e 50–59 anos para as demais DCV, que apresentavam reduções consistentes nas décadas anteriores, registraram aumento no período de 2020–2022, reforçando a hipótese de que a pandemia exerceu influência significativa sobre os padrões de mortalidade cardiovascular seletivamente a determinados grupos populacionais.

Os resultados da Tabela 2, sobre o excesso de mortalidade por subgrupos de DCV no período da pandemia de COVID-19 entre 2020 e 2022, evidenciam um padrão crescente de excesso de mortalidade por subgrupos de DCV durante os anos da pandemia de COVID-19 (2020–2022). Em 2020, observou-se o incremento inicial de 4% no subgrupo outras DCV (SMR=1,04; IC95% 1,03–1,05), enquanto as DCbV e DIC se mantiveram estáveis. Contudo, em 2021, todos os subgrupos registraram excesso significativo: DCbV e DIC apresentaram aumento de 8% (SMR=1,08; IC95% 1,07–1,09), e outras DCV atingiram 15% (SMR=1,15; IC95% 1,14–1,16). Esse padrão intensificou-se em 2022, com taxas de 16% para DCbV (SMR=1,16), 18% para DIC (SMR=1,18) e 23% para outras DCV (SMR=1,23), reforçando a tendência ascendente ao longo do período.

**Tabela 2:** Excesso de mortalidade por subgrupos de doenças cardiovasculares segundo grupo populacional, 2020-2022.

Grupo populacional	2020			2021		
	DCbV	DIC	Outras DCV	DCbV	DIC	Outras DCV
	SMR (IC95%)	SMR (IC95%)	SMR (IC95%)	SMR (IC95%)	SMR (IC95%)	SMR (IC95%)
Masculino	1,01 (1,00–1,02)	0,98 (0,97–0,99)	1,04 (1,03–1,05)	1,07 (1,06–1,08)	1,07 (1,06–1,08)	1,15 (1,14–1,16)
Feminino	1,01 (1,00–1,02)	0,96 (0,95–0,97)	1,04 (1,03–1,05)	1,11 (1,10–1,12)	1,09 (1,08–1,10)	1,16 (1,15–1,17)
0 a 19 anos	0,87 (0,77–0,97)	1,09 (0,92–1,27)	0,88 (0,82–0,94)	0,91 (0,81–1,02)	1,18 (0,99–1,36)	0,89 (0,83–0,95)
20 a 29 anos	0,96 (0,88–1,04)	1,09 (1,01–1,18)	0,95 (0,90–1,01)	1,16 (1,07–1,25)	1,15 (1,07–1,24)	1,04 (0,98–1,10)
30 a 39 anos	1,03 (0,98–1,08)	1,03 (0,99–1,08)	0,97 (0,94–1,01)	1,09 (1,04–1,14)	1,17 (1,12–1,22)	1,04 (1,00–1,07)
40 a 49 anos	0,98 (0,95–1,01)	0,99 (0,96–1,01)	0,98 (0,95–1,00)	1,11 (1,08–1,14)	1,06 (1,04–1,09)	1,09 (1,06–1,11)
50 a 59 anos	1,01 (0,99–1,03)	0,94 (0,93–0,96)	1,02 (1,00–1,03)	1,08 (1,06–1,10)	1,01 (1,00–1,03)	1,11 (1,09–1,12)
60 a 69 anos	0,99 (0,98–1,01)	0,93 (0,91–0,94)	1,01 (0,99–1,02)	1,03 (1,01–1,04)	0,99 (0,98–1,00)	1,10 (1,08–1,11)
70 anos e +	0,98 (0,97–0,99)	0,96 (0,95–0,97)	1,02 (1,01–1,03)	1,04 (1,03–1,05)	1,04 (1,03–1,05)	1,10 (1,09–1,11)
Total	1,00 (0,99–1,01)	0,97 (0,96–0,98)	1,04 (1,03–1,05)	1,08 (1,07–1,09)	1,08 (1,07–1,09)	1,15 (1,14–1,16)
**Grupo populacional**	**2022**					
	**DCbV**		**DIC**		**Outras DCV**	
	**SMR (IC95%)**		**SMR (IC95%)**		**SMR (IC95%)**	
Masculino	1,16 (1,15–1,17)		1,17 (1,16–1,18)		1,23 (1,22–1,24)	
Feminino	1,20 (1,19–1,21)		1,20 (1,19–1,21)		1,23 (1,22–1,24)	
0 a 19 anos	1,06 (0,95–1,17)		1,15 (0,96–1,34)		1,09 (1,02–1,16)	
20 a 29 anos	1,07 (0,99–1,16)		1,35 (1,25–1,45)		1,13 (1,07–1,19)	
30 a 39 anos	1,14 (1,08–1,19)		1,22 (1,17–1,28)		1,11 (1,08–1,15)	
40 a 49 anos	1,14 (1,11–1,17)		1,19 (1,16–1,21)		1,15 (1,13–1,18)	
50 a 59 anos	1,12 (1,10–1,14)		1,11 (1,10–1,13)		1,14 (1,13–1,16)	
60 a 69 anos	1,07 (1,06–1,09)		1,06 (1,04–1,07)		1,13 (1,11–1,14)	
70 anos e +	1,11 (1,10–1,12)		1,11 (1,10–1,12)		1,14 (1,13–1,15)	
Total	1,16 (1,15–1,17)		1,18 (1,17–1,19)		1,23 (1,22–1,24)	

DCbV: doenças cerebrovasculares; DIC: doenças isquêmicas do coração; DCV: doenças cardiovasculares; SMR: razão de moralidade padronizada; IC95%: intervalo de confiança de 95%.

A análise estratificada por sexo revelou que as mulheres tiveram excessos ligeiramente superiores aos dos homens em 2021 (SMR=1,11 *versus* 1,07 para DCbV; 1,09 *versus* 1,07 para DIC) e 2022 (SMR=1,20 *versus* 1,16 para DCbV; 1,20 *versus* 1,17 para DIC).

Quanto à faixa etária, destacaram-se grupos específicos por subgrupo de DCV que tiveram um padrão crescente de excesso de mortalidade. Em 2022, o maior impacto para o subgrupo DCbV foi entre 30–39 e 40–49 anos (ambas as faixas com SMR=1,14) e 50–59 anos (SMR=1,12). Para as DIC, houve pico em adultos de 20–29 (SMR=1,35), 30–39 anos (SMR=1,22) e 40–49 anos (SMR=1,19). Em relação ao subgrupo outras DCV, os indivíduos com ≥40 anos foram os mais afetados com SMR, que variou de 1,13–1,15.

## DISCUSSÃO

Os resultados evidenciaram tendência geral de redução da mortalidade por DCV no Brasil entre 2000 e 2020, com diferenças significativas entre subgrupos, sexos e faixas etárias. Entretanto, em 2020, observou-se alteração nessas trajetórias, caracterizada pela desaceleração da queda e, em alguns casos, inversão da tendência.

Antes da pandemia, o Brasil registrava um declínio contínuo nas taxas de mortalidade ajustadas por idade para DCV, no entanto estudos recentes indicaram que essa tendência foi interrompida ou mesmo revertida durante a pandemia^8^. Dados do Sistema de Informações de Mortalidade revelaram redução de 8,8% nas mortes por DCV entre março e dezembro de 2020, em comparação com o mesmo período de 2019. Essa diminuição pode ser atribuída a subnotificações ou a mudanças nos padrões de registro de óbitos durante a crise sanitária^8^. Além disso, observou-se aumento significativo de óbitos por DCV como comorbidade em pacientes com COVID-19, sugerindo que a infecção pelo Sars-CoV-2 tenha exacerbado condições cardiovasculares preexistentes^9^.

A pandemia afetou de maneira distinta os subgrupos de DCV. As DIC, que incluem condições como infarto do miocárdio, apresentaram redução nas hospitalizações durante os períodos críticos da pandemia. Essa diminuição pode ser resultado do receio da população de procurar atendimento médico por causa do risco de contaminação, levando a atrasos no diagnóstico e tratamento e possivelmente aumentando a mortalidade domiciliar por eventos cardíacos agudos^10^.

No caso das DCbV, como acidentes vasculares cerebrais, também houve redução nas admissões hospitalares. Estudos sugeriram que pacientes com sintomas leves podem ter evitado buscar atendimento imediato, resultando em piora dos desfechos clínicos^10^. Além disso, a sobrecarga dos sistemas de saúde pode ter limitado o acesso a terapias emergenciais, essenciais para minimizar sequelas neurológicas^11^.

O subgrupo de outras DCV, que abrange doenças valvares e miocardiopatias, enfrentou desafios adicionais. Pacientes com condições valvares avançadas, por exemplo, tiveram procedimentos cirúrgicos ou intervencionistas adiados em razão da priorização de recursos para o combate à COVID-19^11^. Essa postergação pode ter contribuído para a progressão da doença e aumento da mortalidade nesse grupo específico^11^.

A análise por faixa etária demonstra que indivíduos mais velhos apresentaram maior vulnerabilidade. Estudos apontaram que pacientes com DCV e COVID-19 tendem a ser mais idosos, com idade média superior à dos pacientes sem DCV^12^. Essa maior suscetibilidade pode ser atribuída à presença de comorbidades e à fragilidade imunológica associada ao envelhecimento^12^. Em relação ao sexo, embora ambos os gêneros tenham sido afetados, algumas pesquisas indicam que homens com DCV podem apresentar taxas mais elevadas de complicações quando infectados pelo COVID-19, possivelmente por causa de fatores hormonais e comportamentais^13^.

A maior suscetibilidade dos grupos etários acima de 40 anos às reversões de tendência na mortalidade por DCV durante a pandemia pode ser explicada por diversos fatores, incluindo a prevalência de comorbidades, os efeitos sistêmicos da COVID-19 e as características inflamatórias e microvasculares associadas tanto ao envelhecimento quanto à própria infecção pelo Sars-CoV-2^11,14^. De fato, a idade avançada é um dos principais fatores de risco para DCV. Indivíduos acima de 40 anos apresentam maior prevalência de condições como hipertensão, diabetes *mellitus*, dislipidemia e obesidade, que são determinantes-chave no desenvolvimento e na progressão das DCV. Durante a pandemia, a presença dessas comorbidades foi amplamente documentada como um fator agravante para a severidade da COVID-19 e para o aumento do risco de desfechos adversos, incluindo morte por DCV^10,15^.

Além disso, o impacto da pandemia sobre esses indivíduos pode ter sido potencializado pela descontinuação do acompanhamento médico regular e pelo atraso no diagnóstico e tratamento de eventos cardiovasculares agudos, como infarto do miocárdio e acidente vascular cerebral^9^. Essa interrupção pode ter contribuído para a piora do controle das doenças crônicas, aumentando a incidência de descompensações cardiovasculares fatais.

Ainda, a tempestade inflamatória induzida pela COVID-19 também desempenha um papel crítico na piora dos desfechos cardiovasculares. O aumento de citocinas pró-inflamatórias, como interleucina-6 (IL-6) e fator de necrose tumoral-alfa (TNF-α), pode desencadear estados de hipercoagulabilidade, contribuindo para eventos trombóticos, incluindo infartos e acidentes vasculares cerebrais^16^. Esse processo pode explicar o aumento da mortalidade por DCV em indivíduos mais velhos, que já têm maior propensão a eventos trombóticos em função de aterosclerose avançada e disfunção endotelial.

As diferenças de mortalidade cardiovascular entre os sexos durante a pandemia também podem ser influenciadas por fatores comportamentais. Estudos pré-pandemia já indicavam que os homens tendem a buscar menos atendimento médico preventivo e têm menor adesão ao tratamento de doenças crônicas, como hipertensão e diabetes, quando comparados às mulheres^17^. Durante a pandemia, essa tendência pode ter sido exacerbada pelo medo de contaminação em serviços de saúde, levando a um maior número de descompensações cardiovasculares e eventos fatais em domicílio.

Em contrapartida, algumas evidências sugerem que as mulheres foram mais impactadas pela interrupção de serviços médicos não emergenciais durante a pandemia, especialmente em relação a consultas de acompanhamento e exames preventivos^18^. Esse fator pode ter afetado de maneira particular as mulheres com DCV prévias, aumentando o risco de desfechos adversos a médio prazo.

Ademais, as diferenças no impacto socioeconômico da pandemia podem ter afetado homens e mulheres de maneiras distintas. A sobrecarga doméstica e a desigualdade no acesso aos serviços de saúde durante o período pandêmico podem ter resultado em desfechos diferenciados entre os sexos, reforçando a necessidade de abordagens personalizadas para mitigar o impacto cardiovascular da COVID-19^19^.

O excesso de mortalidade observado no presente estudo, principalmente em 2021 e 2022, corrobora os achados da análise de série temporal, com magnitudes expressivas que sugerem um fenômeno de *antecipação de mortes* (*harvesting effect*). Esse padrão indica que óbitos que provavelmente ocorreriam em um horizonte temporal mais amplo foram precipitados por fatores interligados ao contexto pandêmico, como a restrição ao acesso a serviços de saúde — incluindo desaceleração de tratamentos, internações e acompanhamentos regulares —, além do agravamento de comorbidades por causa da interrupção de cuidados crônicos. As desigualdades estruturais no acesso à saúde, intensificadas durante a crise, se refletiram no aumento da mortalidade intra-hospitalar por DCV^20^ e na redução de hospitalizações eletivas^9,21^, cenário associado à maior ocorrência de óbitos domiciliares^22^ e à descontinuidade da assistência a pacientes de risco^23^.

Adicionalmente, o isolamento social contribuiu para a elevação de fatores de risco modificáveis, como sedentarismo e estresse, ampliando a vulnerabilidade cardiovascular^24^. Em 2020, observou-se um excesso de mortalidade de 22% por causas naturais no Brasil, com as DCV representando uma significativa parcela desse aumento^25^. O grupo das DCV apresentou excesso de óbitos de 16%^26^, chegando a 77,9% quando consideradas como comorbidade dos óbitos^8^. Apesar de possíveis limitações na qualidade dos registros de mortalidade durante a pandemia, a consistência dos dados reforça a substancial influência da crise sanitária sobre a morbimortalidade cardiovascular, evidenciando não apenas a sobrecarga direta da infecção por Sars-CoV-2, mas também os efeitos indiretos da desorganização dos sistemas de saúde e das mudanças comportamentais.

Ao fim e ao cabo, os dados apresentados corroboram a hipótese de que a pandemia de COVID-19 atuou como um ponto de inflexão nas tendências de mortalidade por DCV no Brasil, afetando diversos grupos etários e ambos os sexos. A interrupção das tendências de declínio prévias, associada ao aumento de óbitos por DCV como comorbidade em pacientes com COVID-19, destaca o impacto significativo da pandemia sobre a saúde cardiovascular da população^8,9^.

Além disso, as diferenças observadas entre os subgrupos de DCV reforçam a segunda parte da hipótese. Enquanto as DIC e DCbV apresentaram reduções nas hospitalizações possivelmente relacionadas ao medo de contaminação e à sobrecarga dos serviços de saúde^12,13^, outras DCV, como doenças valvares, sofreram com o adiamento de procedimentos essenciais, levando a desfechos adversos^13^.

Em resumo, os métodos empregados, baseados em análise de séries temporais e regressão *joinpoint*, permitiram identificar mudanças significativas nas tendências de mortalidade por DCV durante a pandemia de COVID-19 no Brasil. A desaceleração no declínio das DIC e DCbV, bem como o aumento abrupto de outras DCV de 2020 em diante, sugere que os impactos indiretos da pandemia — como a redução no acesso a serviços de saúde, adiamento de procedimentos eletivos e estresse psicossocial — podem ter exacerbado vulnerabilidades preexistentes, especialmente em mulheres e faixas etárias intermediárias (40–59 anos). A metodologia de projeção de óbitos esperados, ancorada em tendências históricas, reforça a robustez da estimativa de excesso de mortalidade (até 23% em 2022), alinhando-se a estudos internacionais^10,11^ que reportaram padrões similares de deterioração cardiovascular durante crises sanitárias.

Em termos de limitações, reconhecemos que a dependência de dados secundários do Sistema de Informações sobre Mortalidade pode implicar erros de diagnóstico, subnotificação de óbitos e inconsistências na codificação de causas de morte, fatores que subestimam a magnitude real da mortalidade por DCV. Além disso, a ausência de informações detalhadas sobre comorbidades e acesso regional diferenciado aos serviços de saúde limita a compreensão de mecanismos causais específicos. Apesar dessas restrições, os resultados oferecem uma visão coerente e relevante da dinâmica da mortalidade cardiovascular durante a pandemia, destacando padrões críticos como o aumento expressivo de outras DCV e a vulnerabilidade de grupos etários intermediários.

Esses achados reforçam a necessidade de políticas públicas que garantam a continuidade do cuidado a pacientes crônicos durante crises, além de investimentos em sistemas de vigilância mais precisos para capturar nuanças regionais e diagnósticas. A estratificação por sexo e idade, ainda que sujeita às limitações dos dados, evidencia disparidades que demandam intervenções focalizadas, consolidando o estudo como uma base empírica crucial para orientar estratégias de mitigação em futuros cenários de emergência sanitária.
